# Valence of Facial Cues Influences Sheep Learning in a Visual Discrimination Task

**DOI:** 10.3389/fvets.2017.00188

**Published:** 2017-11-06

**Authors:** Lucille G. A. Bellegarde, Hans W. Erhard, Alexander Weiss, Alain Boissy, Marie J. Haskell

**Affiliations:** ^1^Scotland’s Rural College, Edinburgh, Scotland; ^2^UMR Modélisation Systémique Appliquée aux Ruminants, INRA, AgroParisTech, Université Paris-Saclay, Paris, France; ^3^School of Philosophy, Psychology and Language Sciences, The University of Edinburgh, Edinburgh, Scotland; ^4^UMRH, INRA, Vetagro Sup, Lyon, France

**Keywords:** sheep, faces, emotions, discrimination task, ovis aries, cognition

## Abstract

Sheep are one of the most studied farm species in terms of their ability to process information from faces, but little is known about their face-based emotion recognition abilities. We investigated (a) whether sheep could use images of sheep faces taken in situation of varying valence as cues in a simultaneous discrimination task and (b) whether the valence of the situation affects their learning performance. To accomplish this, we photographed faces of sheep in three situations inducing emotional states of neutral (ruminating in the home pen) or negative valence (social isolation or aggressive interaction). Sheep (*n* = 35) first had to learn a discrimination task with colored cards. Animals that reached the learning criterion (*n* = 16) were then presented with pairs of images of the face of a single individual taken in the neutral situation and in one of the negative situations. Finally, sheep had to generalize what they had learned to new pairs of images of faces taken in the same situation, but of a different conspecific. All sheep that learned the discrimination task with colored cards reached the learning criterion with images of faces. Sheep that had to associate a negative image with a food reward learned faster than sheep that had to associate a neutral image with a reward. With the exception of sheep from the aggression-rewarded group, sheep generalized this discrimination to images of faces of different individuals. Our results suggest that sheep can perceive the emotional valence displayed on faces of conspecifics and that this valence affects learning processes.

## Introduction

Faces are an essential source of information for social species ranging from primates to ungulates such as sheep. By looking at the face of another animal, individuals can obtain information about identity, emotional state, sexual attraction, or gaze direction (1). Sheep are one of the most studied livestock species in terms of face processing and are able to discriminate between faces of at least 50 conspecifics, and to remember these faces for up to 2 years (2). Sheep, like cattle, are also sensitive to social familiarity in faces and show preferences for familiar faces over unfamiliar ones (3, 4). Individual recognition based on faces is also stable over time in sheep; ewes trained to identify images of faces of 3-month-old lambs were able to discriminate the same lambs aged only 1 month (5).

In animals, emotional states can be expressed through vocalizations (6), odors (7), posture (8), or facial expressions (9). Outward expressions of emotions are a way of communicating social information to conspecifics as well as across species, as highlighted in recent studies of perception of human faces by dogs, horses, and even giant pandas (10–12). Despite their relative lack of facial mobility or of a facial musculature as complex as that of non-human primates, sheep display emotional expressions through their faces, and especially through ear postures (13, 14). The role of facial features such as eyes, mouth, and cheek muscles have also been identified in sheep facial expressions linked to pain (15). Moreover, conspecifics can distinguish between facial displays of emotions. Indeed, when presented with images of the face of the same familiar conspecific taken in a stressful (isolation) or in a calm situation, sheep showed a preference for the calm face (3).

Animals thus possess a wide variety of ways to express their emotions. For researchers in the field of animal welfare science, being able to assess animals’ emotional states has been a major focus, moving from more traditional physiological and behavioral measures to the development of the cognitive bias tests (16). Cognitive bias refers to the influence that the valence of emotional states has on cognitive processes, leading to biases in judgment, memory, or attention (17). For instance, animals in negative emotional states make “pessimistic” judgments in judgment bias tests, while animals in positive emotional states show “optimistic” judgments. This method has been applied to several species, and has especially been used to assess the impact of husbandry practices on the welfare of farmed species [reviewed in Ref. (18)]. Most recently, judgment bias tests have even been extended to insects (19, 20).

The present study was part of a larger project investigating the potential use of images of faces of conspecifics as cues in judgment bias tests with small ruminants. Its first aim was to assess the ability of sheep to distinguish between facial displays of different emotional states. To that end, we first investigated whether sheep could learn to use images of faces of familiar conspecifics displaying different emotional states as cues in a simultaneous discrimination task. We took photos of sheep in three situations (social isolation, aggressive interaction, and ruminating in the home pen) that are considered to induce emotional states of different valence. In the first phase of training, we used simple colored cards, to ensure sheep could learn the discrimination task in the experimental setup. In the second phase, sheep were trained with pairs of images of faces taken from the same individual but in two different situations.

Since most cognitive bias studies use secondary reinforcers, an extensive training phase is required whereby animals learn to associate one cue with a positive consequence and another cue with a negative consequence. If the valence of the emotional state experienced by a sheep can be perceived by conspecifics in images of its face, then faces would be stimuli with an inherent value for the other animals observing them, and thus the training phase would not be necessary. Hence, the second aim of this study was to determine whether sheep perceived the valence of the emotional state displayed in an image of the face of a conspecific. Social familiarity has been shown to influence learning speed in discrimination tasks, with sheep learning to discriminate faster between faces of a familiar breed than between faces of an unfamiliar breed or between symbols (21). However, little is known about the influence of facial expressions of different emotional states on the learning process in a discrimination task. We hypothesized that learning speed is affected by the type of images rewarded, i.e., that the emotional valence displayed in the image of a face affects learning, but that during the first training phase with colored cards, the type of cards rewarded would not affect learning speed. Presenting images of faces has been shown to reduce stress in sheep (22) and images of conspecifics are primary reinforcers, i.e., they are naturally approached by sheep (23). We thus predicted that learning the association between an image of a neutral face and a reward but also generalizing this association to images of faces of new familiar individuals would be easier than the association between the image of a stressed face and a reward.

## Materials and Methods

### Ethical Note

All experimental procedures were approved by the Scotland’s Rural College (SRUC) Edinburgh Animal Ethics Committee (Protocol no. ED-AE-2-2014). Animals were closely monitored before, during, and after the study.

### Animals and Housing

Testing took place between March and July 2014 at the SRUC Woodhouselee experimental farm at Easter Bush (UK). Forty non-pregnant female Scottish Mule sheep of 10–12 months of age (37.1 ± 4.8 kg) were used in this study. The sheep were born and reared on the experimental farm and were familiar with each other, having lived in the same flock for at least 6 months prior to the study. Four sheep (thereafter referred to as Photo Sheep) were pseudo-randomly selected based on body weight and had their faces filmed. This selection on body weight enabled us to select animals that were of average body weight (37.55 ± 3.6 kg). The positive correlation between live weight and hierarchy is well established in ungulates (24, 25) and choosing sheep of intermediate weight was done to avoid the selection of only dominant animals. The Photo Sheep did not take part in the discrimination task but were housed with the rest of the group until the end of the study.

All sheep were housed indoors in a straw-bedded pen (4 m × 12 m) for the duration of the experiment. Animals had *ad libitum* access to hay and water as their main diet. They were also fed a limited amount of concentrate pellets (0.5 kg per animal per day) after training every day and showed a high motivation to eat it. This allowed us to use concentrate pellets as reward in the tests without having to food-deprive the animals.

### Habituation to Handling

Scottish Mules sheep are a hill breed and typically have limited contact with humans throughout the year. The experimental animals had little experience of human handling and living indoors, and therefore underwent a short phase of systematic desensitization to facilitate handling (26), and to limit the impact of handling stress on responses to tests. This habituation procedure involved four consecutive steps that allowed the animals to gradually adapt.

First, the animals were handled in three smaller groups (two groups of 13 and one group of 14 sheep). A group was only moved to the next step once all sheep went calmly through the previous step. For the first step, the group was moved into a small handling pen with the gate open. For the second step, the group was confined in a small handling pen with no human handler present. The third step consisted of confining the group in the same small handling pen, but with an experimenter standing just outside the pen. Finally, for the fourth step, the experimenter had to touch calmly every sheep within the group. Following the fourth step, each group of sheep was moved through raceways into 4 m × 4 m pen that served as the test arena. Once in the test arena, they remained there for 10 min and received a small amount of concentrate feed. This manipulation was repeated three times with the group size decreasing to five and then to two sheep.

### Images of Faces

**Figure 1:**
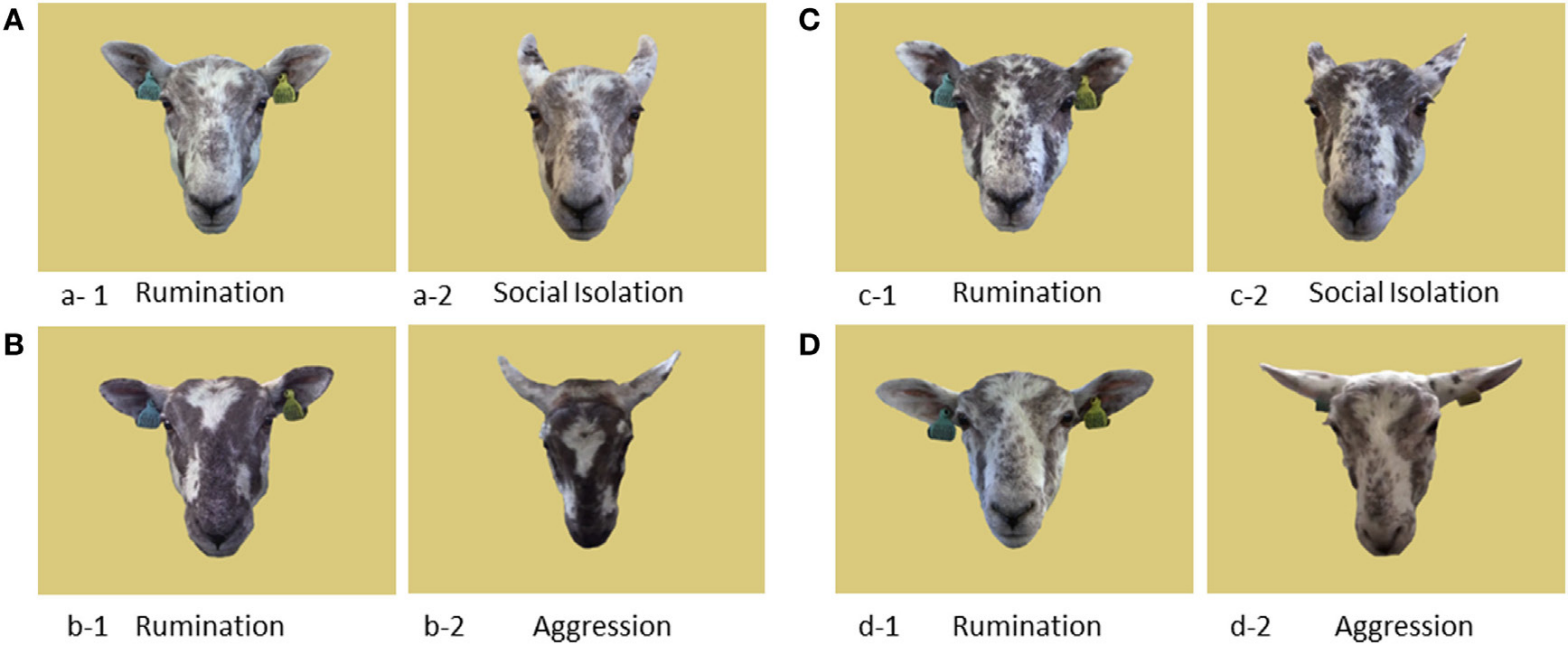
Pairs of images obtained from four different Photo Sheep **(A–D)** and presented simultaneously in the maze during training and test sessions. **(A,B)** were used during training and **(C,D)** during tests.

We filmed each of the four Photo Sheep in three situations. For each situation, short video clips of the Photo Sheep were taken with an HD camcorder (Legria HFM52, Canon, Tokyo, Japan) and frames with a full clear frontal view of the face were extracted from the video clips using Pinnacle Studio 17 (Pinnacle Systems, 2013). Then, using Adobe Photoshop CC (Adobe Systems, 2014), the faces were digitally cut from the frames and placed against a neutral beige background (RGB model: *R* = 217, *G* = 202, *B* = 126) and levels of brightness and contrast were adjusted (Figure 1).

#### Ruminating in the Home Pen

The Photo Sheep were filmed by a familiar experimenter while standing ruminating in their home pen. Rumination was considered to be a relaxed state of neutral valence and low arousal, similar to the one described in horses by Wathan et al. (27). Rumination has also been used as an indicator of habituation to a stressful situation in sheep (28). Animals had their ears in the frontal plane, showed no flared nostrils or wide eyes and were looking straight at the camera (Figure 1, a-1, b-1, c-1, d-1).

#### Social Isolation

**Figure 2:**
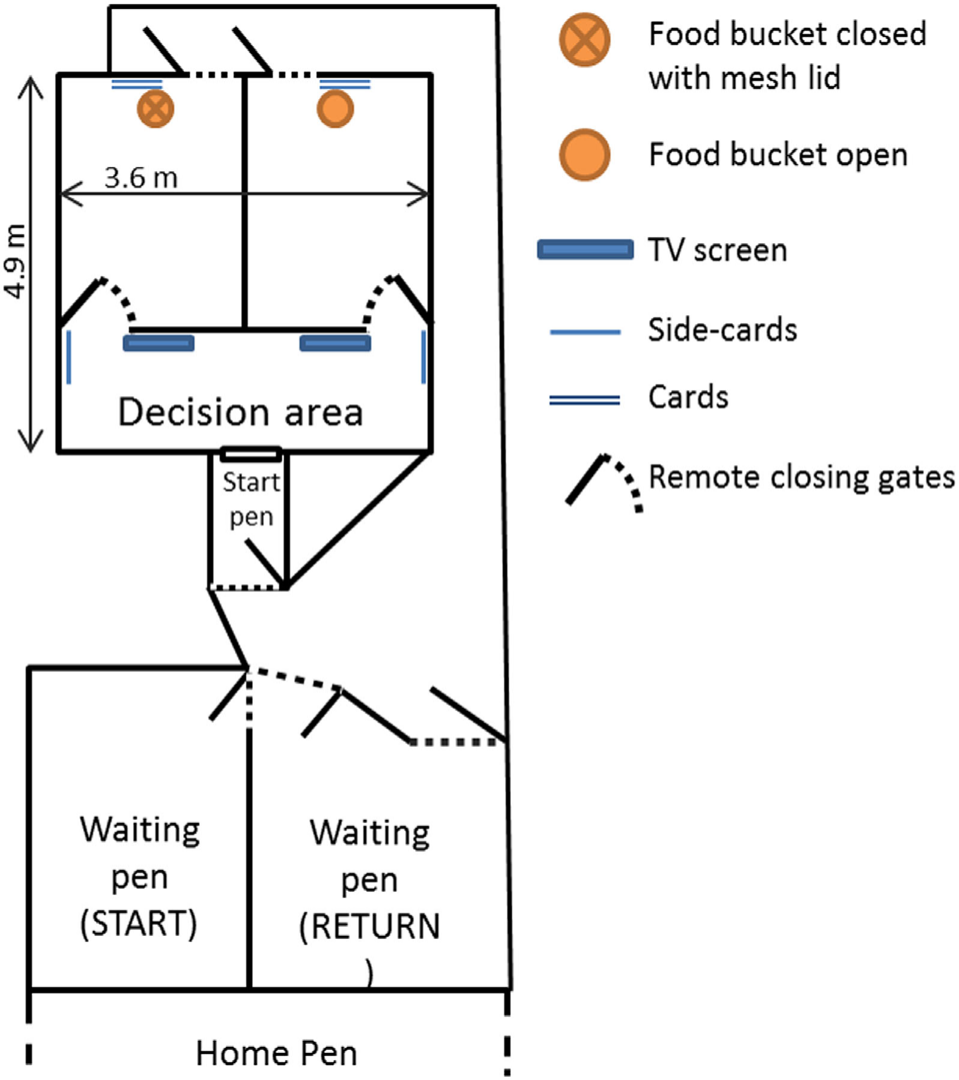
Representation of the experimental setup. This includes the two-armed maze, the start pen and the start and return waiting pens, as well as the raceways connecting them. The position of the food buckets alternated between runs depending on which side the rewarded image was placed. “Side cards” and “cards” represent the cards where the cues displayed on the screens were repeated on laminated printed A3 sheets (approximately the size of the screen). The gates leading to the two arms of the maze could be closed remotely.

Each of the Photo Sheep was isolated in a small pen (4.5 m × 4.5 m) with solid walls (approximately 140 cm high) for 90 s. The Photo Sheep were only isolated once. No visual contact with conspecifics was allowed, but the pen was located in the same building as the home pen, and so auditory and olfactory contact with other sheep was maintained. Short video clips were recorded by two hidden experimenters. All animals displayed stress-related behaviors such as increased locomotion, high pitched vocalizations (23, 29), and attempts to escape from the test pen (30). This situation was thus considered as inducing an emotional state of negative valence and high arousal (Figure 1, a-2, c-2).

#### Aggressive Interactions

A trough allowing access to concentrate feed to only one sheep at a time was placed in a test arena with solid walls (4.5 m × 4.5 m). Photo Sheep were paired for this situation and all possible pairs were filmed (six pairs). A given pair of Photo Sheep entered the test arena simultaneously and was given 2 min to interact while being filmed by two hidden experimenters. In each pair, both Photo Sheep showed agonistic behaviors such as head threats, head butts, or pushes (29, 31). Images of faces were created from faces of Photo Sheep filmed frontally and while initiating a bout of aggressive interaction (head threat) (Figure 1, b-2, d-2), and this situation was considered to have induced a negative emotional state of high arousal in both sheep.

### Discrimination Task in a Two-Armed Maze

**Figure 3:**
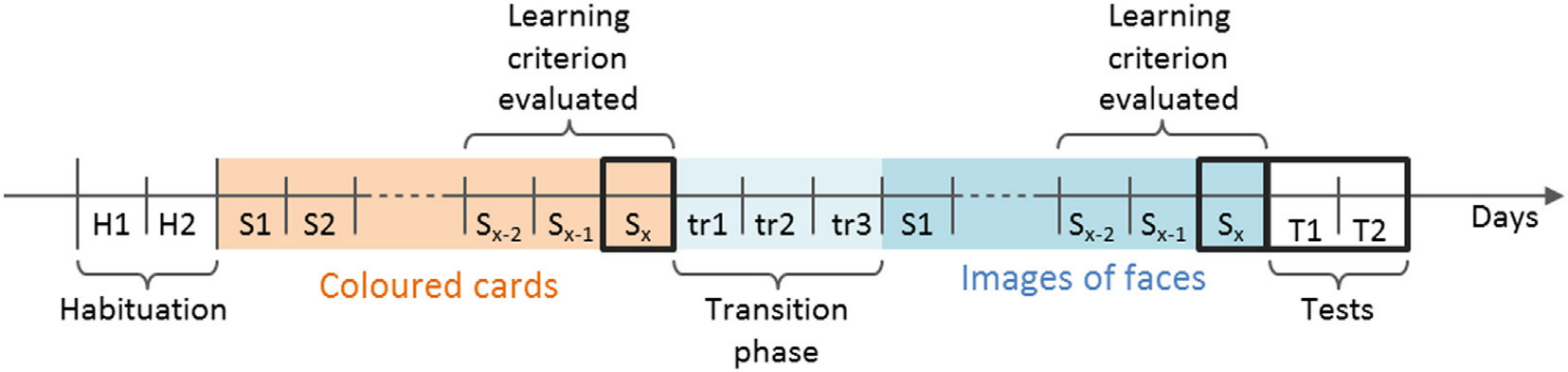
Timeline of the five consecutive phases of the study. The total number of sessions needed to reach the learning criterion varied between animals (noted *x*), for Colored Cards, *x* ≤ 18, for Images of faces *x* ≤ 15. The learning criterion (≥80% correct choices over two consecutive sessions, or ≥80% correct choices in two out of three consecutive sessions and ≥60% in the penultimate) was evaluated during the three last sessions of both the Colored Cards and the Image of faces phase, as indicated. Sessions framed by thick black lines were analyzed. During transition sessions, images of faces framed by the corresponding color (rewarded image = rewarded shade of green and *vice versa*) were presented. The final training sessions are noted *S_x_*. H, habituation; S, training sessions; T, tests sessions; tr, transition sessions.

In the simultaneous discrimination task, sheep had to learn to associate one cue with a food reward and a second cue with a negative consequence. Positive reinforcement consisted of a food reward, namely a small amount of concentrate pellets (12.5 ± 1.5 g) placed in a bucket. Positive punishment consisted of keeping the sheep in social isolation for 60 s in the incorrect arm of the maze. A bucket containing pellets but closed with a mesh lid was also placed in the incorrect arm, so that the animal could see and smell but not eat the food (Figure 2). The type of cue used depended on the training phase. There was no previous evidence of sheep learning a discrimination task either in a similar setup or with images of faces. Consequently, if sheep had failed to learn the task with images of faces, it would have been impossible to distinguish whether this failure was due to the discrimination task being too complex, or because it was too difficult for the animals to process the new type of cues. Thus, before images of faces, simple colored cards were used as cues, to determine whether sheep were capable of learning the simultaneous discrimination task. Using cards in the first phase also allowed us to compare sheep’s behavioral responses when presented with neutral stimuli and with images of faces. The experiment was divided into five consecutive phases: (1) habituation to the maze, (2) training with colored cards, (3) transition training, (4) training with images of faces, and (5) tests with new images of faces (Figure 3).

#### Experimental Setup

The discrimination task took place in a two-armed test maze (4.8 m × 3.6 m) with solid wooden walls (Figure 2). At the beginning of a trial, sheep were moved from the home pen into a waiting pen (labeled “START,” Figure 2) which was connected to a start pen that gave access to the maze through a sliding door. A 2.5-m long wooden wall was placed at 1.9 m from the entry gate, with two open gates leading to the two arms of the maze. These gates could be closed remotely by an experimenter standing outside the maze once a sheep had entered one of the arms. This wall also supported two flat computer screens (48 cm diagonal), one at each end near the gates, on which the two cues were simultaneously displayed. The cue displayed on each screen was also shown twice in each corresponding arm: on a card hanging on the wall next to the remotely closing gate (hereafter referred to as side-card) and on another card placed on the rear wall of each arm. The area between the entry gate and the wall with the screens was referred to as the decision area, i.e., the area where the sheep had to choose between the two arms of the maze. Both arms of the maze had an exit door opening onto a raceway leading back to the “return” waiting pen that was adjacent to the home pen.

#### Habituation to the Experimental Setup

The habituation phase was divided into three steps over 2 days (Figure 3). Sheep were considered to be habituated once they no longer displayed a stress response while being handled or in the test-pen. Due to the preliminary phase of systematic desensitization to human handling, habituation to the experimental setup was fairly short. On the first day, the sheep visited the maze in randomly allocated groups of three and were allowed to explore it for 3 min (all gates remained open). This was repeated three times consecutively per triad in total. The sheep were then randomly split into pairs and entered the maze three times consecutively for 2 min. On the second day of the habituation phase, sheep were brought into the maze individually for 1 min. Again, this was repeated three times consecutively for each individual. One sheep had to be removed from the study at that stage due to health issues, and thus 35 sheep were included in the next phase of the experiment.

#### Training Phases

##### First Training Phase: With Colored Cards

Two shades of green differing in tone and brightness were used as cues for the first training phase (light: Red = 240, Green = 241, Blue = 223; dark: Red = 122, Green = 188, Blue = 50). Sheep can easily distinguish shades of green that differ only in brightness (32). The chosen cues also differed slightly in tone and so ensured that a good contrast between the two colors would be maintained on the screens.

For half of the tested sheep, light green was the rewarded cue and dark green was the punished cue. The other half of the group received the opposite pairings. The rewarded side alternated following Gellerman series (33) to prevent the sheep from place learning. For the first eight runs in the maze, one of the remote-closing gates was closed prior to the sheep’s entry. This preliminary conditioning session forced the animal to explore each possible side/reward combinations and their consequences (incorrect-left, correct-right, incorrect-right, and correct-left) twice. Side and type alternated, starting with incorrect-left, so that the final run was forced-rewarded. These preliminary conditioning runs in the maze were not taken into account in the analyses.

A training session then consisted of 10 consecutive runs through the maze. The order of the side/reward combination also followed Gellerman series (33) and was changed after each session to prevent the sheep from learning the order or developing a side bias. The outcome (side chosen and success or failure) was recorded for each run.

For the first 7 training sessions, since all 35 sheep could not be trained in 1 day, the group was split into two groups of 17 and 18 sheep. Each group went through a training session every other day. After the seventh training session, five animals that had shown consistent side biases (i.e., animals that consistently chose the same side for every run through the maze) were removed from the study. As a result, all animals then trained every day. A sheep reached the learning criterion once it had reached a minimum of 80% correct responses in two consecutive sessions, or 80% of correct choices in two out of three consecutive sessions and at least 60% correct choices in the penultimate session (i.e., ≥80%, ≥60%, ≥80%). This is equivalent to a minimum of 22 correct choices out of 30 consecutive runs, or a minimum of 73% of correct answers across 3 consecutive sessions. If after 18 training sessions the animals had still not reached the learning criterion, they were excluded from the next phase of the study.

##### Second Training Phase: With Images of Faces

In this phase of training, colors were replaced with images of the faces of the Photo Sheep (Figures 1A,B). A pair of cues consisted of two images of the same Photo Sheep: one taken in the neutral situation and one in one of the two negative situations (SI for social isolation or Aggr for aggressive interactions). To differentiate the neutral image paired with SI from the neutral image paired with Aggr, neutral images from SI-Neutral pairs are referred to as N_SI_ and neutral images from Aggr–Neutral pairs are referred to as N_Aggr_. The type of rewarded image was attributed alternatively to each sheep that reached the learning criterion in the first phase (*n* sheep). The four types of images (SI, Aggr, N_SI_, and N_Aggr_) were allocated so that for half of the test sheep (*n*/2 sheep) the correct cue was an image from one of the negative situations (SI, *n*/4; Aggr, *n*/4), and for the other half, the correct cue was an image from the neutral situation (N_SI_, *n*/4; N_Aggr_, *n*/4). A given sheep was trained with images of the face of the same individual.

Each sheep went through three transition training sessions to facilitate the transfer of the colored card cues to the facial cues. For the first session (tr1, Figure 3), each face was framed by the color sharing the same attributes, i.e., the now rewarded face was framed by the color previously rewarded and *vice versa*. The color was also repeated on the side-card, and the card placed above the bucket in the arm was a repetition of the framed face. For the next two sessions (tr2 and tr3, Figure 3) the colored side-card was removed but the pictures were still framed in shades of green. These three transition training sessions were not included in the number of sessions needed to reach the learning criterion, as the aim of the experiment was to test the ability to learn to identify facial expressions, and not a combination of colored cards and expressions.

After the three transition sessions, the only cues available to the sheep to choose an arm of the maze were images of faces presented on the screens and repeated on the cards above the feed bucket (Figure 2). The learning criterion was the same as during the colored cards phase. As soon as a sheep had reached the learning criterion, it was moved to the test phase.

#### Test: Generalization to Images of New Familiar Individuals

The test phase consisted of two sessions of ten runs each, where the images presented to the test sheep were of the face of a different Photo Sheep. The Photo Sheep used in this test phase were also familiar with the test sheep, but images of their faces had never been presented in the maze (Figures 1C,D). The test sheep had to generalize the task they had learned to images of new familiar individuals to gain access to the food reward. The type of rewarded image did not change during this phase, e.g., sheep that had learned to associate SI images with a reward had to associate SI images of a new Photo Sheep with the reward.

### Data Collection and Statistical Analysis

For both training phases (colored cards and images of faces), learning speed, i.e., the number of sessions needed to reach the learning criterion, was recorded for each sheep. For every run of the training and test phases, the outcome (success or error), the time from the sheep’s entry into the maze (two front feet inside the maze) to its choice (gate arm closed behind the sheep) were recorded (LatChoice, seconds) from video files using The Observer 5.0 (Noldus Information Technology, Netherlands).

All analyses with non-parametric tests were conducted in Minitab 17 (Minitab Inc., PA, USA). Mixed models were run in GenStat 16th edition (VSN International Ltd., UK). Significance level was set at *P* = 0.05.

Due to the small number of animals (16 sheep were included in the second training phase, four for each type of image rewarded, see Section “Results” for more details), the two types of negative images (SI and Aggr) and the two types of neutral images (N_SI_, when the second image was SI, N_Aggr_ when the second image of the pair was Aggr) were grouped under “negative images” and “neutral images,” respectively, for analysis of learning speed. The four types of images were not grouped in other analyses. Data were tested for outliers using Grubb’s test at a 5% level of significance and differences in learning speed between the categories of rewarded images were then analyzed by Mood’s median tests, which are more robust than Kruskal–Wallis tests against outliers (34).

All sheep needed a different number of training sessions to reach the learning criterion, so for a given sheep the final training session of a phase did not necessarily have the same session number than for another sheep (e.g., the final training session could be Session 3 for one individual, and Session 14 for another). However in this final training session, all sheep were at a similar state of training and understanding of the task (at least 80% correct choices). Thus, the final training sessions of both training phases were analyzed (Figure 3, framed in black). The two test sessions, where sheep had to generalize the task, were also analyzed.

For the selected training and test sessions, the effect of the type of rewarded image (N_Aggr_, N_SI_, Aggr, or SI) on the success of a run (0 or 1) was analyzed by a generalized linear mixed model (GLMM) using a binomial distribution and logit link function. The type of rewarded image (N_Aggr_, N_SI_, Aggr, or SI) was included as a fixed effect in the final model, and Animal and Session were included as random effects, with Session nested within Animal.

If sheep chose at chance level during a test session, then the mean value of the success variable was 0.5. In that case, with the logit transformation used by the GLMM the mean would be as follows: logit (0.5) = ln (0.5/(1−0.5)) = 0. To test whether sheep chose the correct image at above chance levels during the two test sessions, the confidence interval (CI) was calculated for the mean value of the success variable for each type of rewarded image (N_Aggr_, N_SI_, Aggr, or SI), based on the output from the GLMM analysis. If the CI included 0, it was not possible to tell whether the sheep had chosen above chance level.

LatChoice was transformed using a natural log function to conform to statistical assumptions. For the same selected training and test sessions (Figure 3), LatChoice was then analyzed by restricted maximum likelihood with repeated measurements, using a power model to account for correlations within subjects across time. Type of rewarded image (N_Aggr_, N_SI_, Aggr, or SI), outcome of the run (success or error), and their interactions were included as fixed effects. Random effects included Run and Animal. *Post hoc* pairwise comparisons were conducted using least significant difference tests. Normality of the residuals was checked graphically.

## Results

### Training with Colored Cards

**Figure 4:**
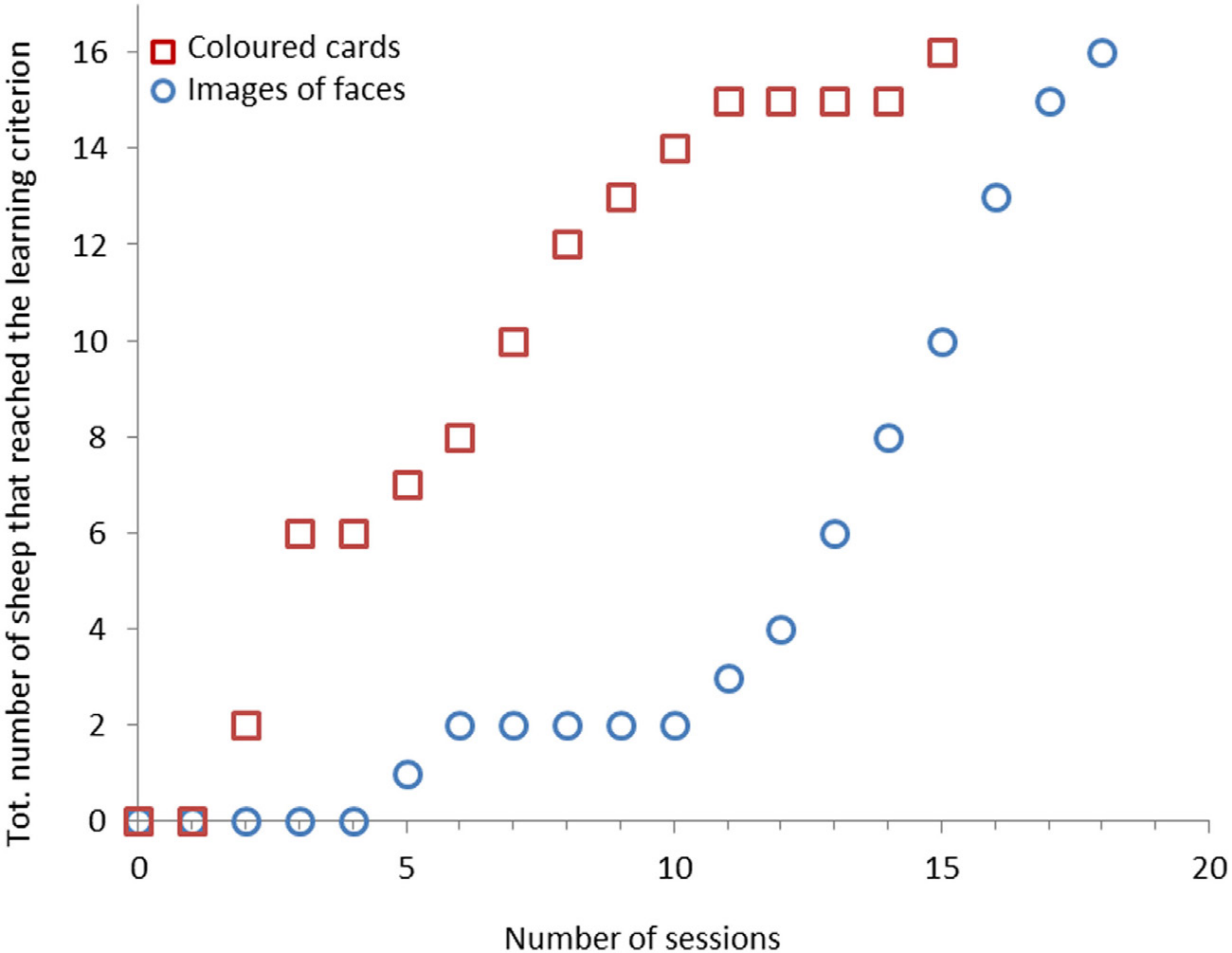
Cumulative number of sheep (*n* = 16) that reached the learning criterion for each session. Training phase with colored cards is coded with blue circles and training phase with images of faces with red squares.

Sixteen sheep (46%) reached the learning criterion in 18 sessions or fewer. Only two sheep learned the task in fewer than 10 sessions (7% of total number of sheep or 12.5% of successful sheep) (Figure 4). There was no difference in learning speed between sheep that had to associate a light green card with the reward and sheep that had to associate a dark green card with the reward (Medians: 14 vs. 15 sessions, χ^2^ = 0.25, *df* = 1, *P* = 0.614).

There was no effect of the type of colored card rewarded (dark green or light green, *F*_1,37.7_ = 0.28, *P* = 0.598), Success (correct vs. incorrect choice, *F*_1,128.9_ = 0.17, *P* = 0.683), or the interaction between those two factors (*F*_1,131.2_ = 0.80, *P* = 0.373) on LatChoice.

### Training with Images of Faces

All 16 sheep (100%) reached the learning criterion with images of faces within 15 training sessions. Fifteen of these animals (94%) reached the learning criterion after 11 sessions (Figure 4).

**Figure 5:**
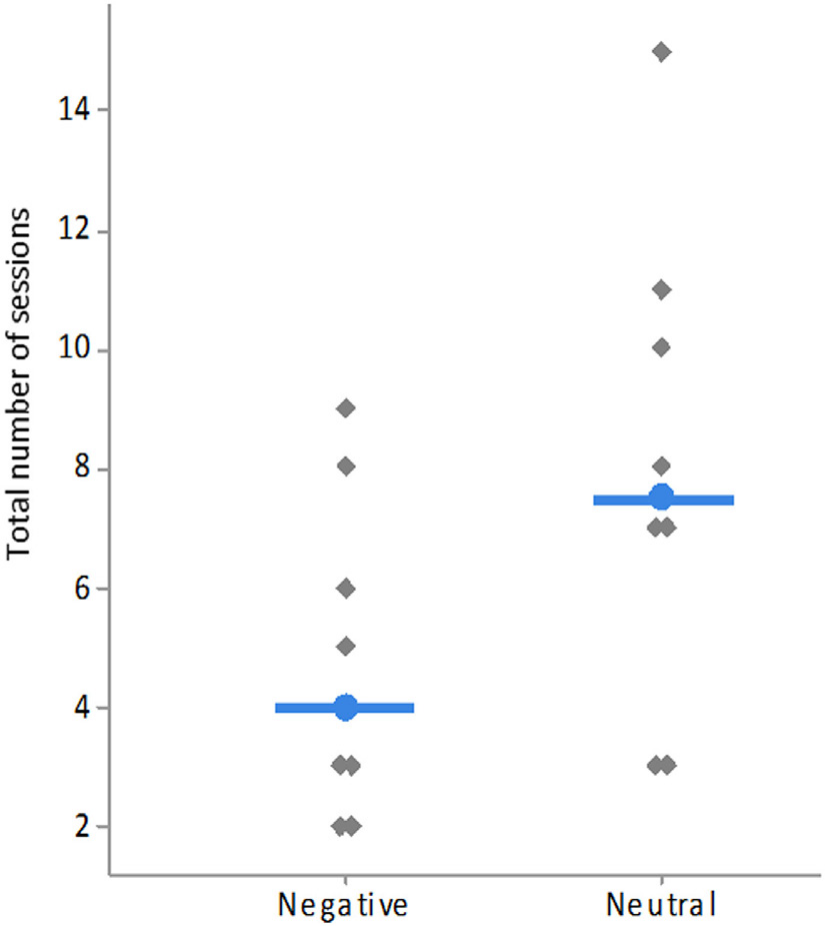
Total number of sessions needed to reach the learning criterion by type of rewarded image (neutral = N_Aggr_ and N_SI_, negative = Aggr, and SI). Each gray lozenge represents one individual sheep. Medians of each group are indicated by the blue bar and dot.

There was a significant difference in learning speed between sheep that had to associate a neutral image (N_Aggr_ or N_SI_) with the reward and sheep that had to associate a negative image (SI or Aggr) with the reward. Sheep learned the task faster (i.e., needed fewer training sessions) when a negative image was rewarded (Medians: 4 vs. 7.5 sessions, χ^2^ = 4.00, *df* = 1, *P* = 0.046, Figure 5). Grubbs’ test results showed that there were no outliers (*G* = 1.17, *P* > 0.90).

In the final training session, there was no effect of the type of image rewarded on the sheep’s number of correct choices (Aggr: 9 ± 0.82, SI: 9.5 ± 1.0 N_Aggr_: 8.25 ± 0.5, N_SI_: 8.5 ± 0.58; *F*_3,153_ = 0.83, *P* = 0.477). There was no effect of the type of image rewarded (N_Aggr_, N_SI_, Aggr, or SI, *F*_3,34_ = 0.23, *P* = 0.879), Success (*F*_1,124.9_ < 0.01, *P* = 0.949), or the interaction between those factors (*F*_3,124.7_ = 0.23, *P* = 0.879) on LatChoice either.

### Tests: Generalization to Images of New Familiar Individuals

The generalization of the task to pairs of images of faces of new familiar individuals was affected by the type of rewarded image (N_Aggr_, N_SI_, Aggr, or SI, *F*_3,26.8_ = 3.43, *P* = 0.031). Based on the CIs, sheep that had Aggr as their rewarded image did not choose the correct image at above chance levels, while sheep that had SI, N_Aggr_, or N_SI_ as their rewarded image did (Table 1).

**Table 1:** CI of the mean number of successes by type of image rewarded (N_Aggr_, N_SI_, Aggr, or SI).

	Mean	CI lower bound	CI higher bound	Backtransformed mean (mean % of success)
Aggr	−0.052	−0.654	0.551	49%
SI	0.754	0.32	1.376	68%
N_Aggr_	1.206	0.538	1.875	77%
N_SI_	1.137	0.475	1.798	76%

*To test whether sheep chose the correct image at above chance levels during the two test sessions, the CI was calculated for the mean value of the success variable for each type of image rewarded, based on the output from the GLMM analysis. If sheep chose at chance level during a test session, then the mean value of the success variable was 0.5, so with the logit transformation used by the GLMM the mean was as follows: logit (0.5) = ln (0.5/(1−0.5)) = 0. So if the CI included 0, it was not possible to conclude that the sheep chose above chance level. Transformed CI and means are presented, and backtransformed means are included to help with interpretation. CI, confidence interval; GLMM, generalized linear mixed model*.

LatChoice was significantly higher when sheep made the correct choice than when they made a mistake (correct choice: 9.4 ± 7.8 s, wrong choice: 7.9 ± 10.8 s; *F*_1,292.9_ = 13.26, *P* < 0.001) but the type of image rewarded (N_Aggr_, N_SI_, Aggr, or SI, *F*_3,12.1_ = 1.10, *P* = 0.385) or its interaction with Success (*F*_3.297.9_ = 0.40, *P* = 0.756) had no effect on LatChoice.

## Discussion

We investigated whether sheep could discriminate between images of faces of familiar conspecifics taken in situations eliciting emotional states of neutral or negative valence, using a simultaneous discrimination task in a two-armed maze. We also assessed the influence of the valence of the rewarded image on learning and generalization processes. All sheep that learned the preliminary discrimination task with colored cards reached the learning criterion with images of faces. There was no difference in learning speed between the two shades of green of the colored cards; however, sheep learnt to associate the food reward with a negative image faster than with a neutral image.

### Influence of the Type of Images of Faces on Learning Processes

As predicted, the type of image rewarded (neutral vs. negative) had an effect on the learning process, while the type of colored card rewarded (light or dark green) did not. However, we observed the opposite of our hypothesis regarding learning speed. Images of calm conspecifics are approached voluntarily by sheep (3) and can therefore be considered as primary reinforcers. We had originally proposed that sheep learning the association between images of ruminating Photo Sheep and food reward would reach the learning criterion faster than sheep learning the association with images taken in negative situations. However, sheep actually learned the task more quickly when their rewarded image was one of the two negatives images (SI or Aggr). It is of course possible that sheep learned to avoid the unrewarded image rather than to approach the rewarded one; however, this would not impact our findings regarding the ability of sheep to discriminate between images of faces.

In humans, negative stimuli (colored images of beetles, negatively valenced images from the International Affective Picture System database) induce stronger and faster responses (e.g., higher amplitude and shorter latency of electrophysiological markers, shorter response time in key pressing) than positive or neutral stimuli (colored images of buildings, neutral or positively valence images from the International Affective Picture System database (35, 36)). Similarly, in flash suppression studies of the perception of facial expressions, fearful expressions have been shown to gain access to awareness more quickly than neutral or happy expressions (37). In animals, a previous study showed that sheep were indeed more attentive (head turned to the screen for at least 2 s) toward videos showing agonistic interactions between conspecifics than toward videos showing ruminating sheep (38). Goats have also been shown to be more attentive toward images of faces of conspecifics photographed in a negative situation (ice pack applied to the udder) (39). From an evolutionary point of view, it is appropriate for animals to pay more attention to faces displaying negative emotions as they could signal the presence of potential threats. In our study, if the attention of sheep was increased toward negative images of faces, this may have aided them to learn to associate an image of a negative face and reward faster. The difference in learning speed could thus be initial evidence that sheep can not only distinguish between facial features, but that they also perceive the valence of the expression shown on the images. In this way, sheep would perceive images of faces taken in negative situations as at least as more interesting, but potentially as negative. This would represent a first step toward the use of images of faces in cognitive bias studies. Further studies are needed to determine if the differences in learning performances were due to sheep paying more attention to negative images, or if images taken in situations of high arousal but positive valence would have the same effect.

The generalization of the discrimination task to images of faces of new familiar individuals during tests was also affected by the type of image rewarded (N_Aggr_, N_SI_, Aggr, or SI). Only Aggr-rewarded sheep were not able to generalize the task to images of new familiar individuals. Since sheep that had N_Aggr_ as their rewarded image had no difficulties in generalizing the task, the poorer results from the Aggr-rewarded group cannot be explained by an increased difficulty in discriminating the neutral from the aggressive face in the new pair of cues. SI- and Aggr-rewarded sheep had reached the learning criterion faster. Consequently, these sheep had been exposed less often to images of faces than Neutral-rewarded sheep. Having a greater experience of the images might have helped the latter to be better at generalizing the task to new images. However, SI-rewarded sheep could generalize the task to new images and did not differ from Neutral-rewarded sheep in their ability to generalize. Therefore, the previous experience of images of faces cannot entirely explain the poorer performance from the Aggr-rewarded animals.

It is also possible that the identity of the Photo Sheep influenced the results from the Aggr-rewarded sheep. If the new Photo Sheep was a very dominant animal, seeing it presenting an aggressive expression might have prompted a strong avoidance response. However, we selected Photo Sheep so that they would be of average body weight since the positive correlation between live weight and hierarchy has been established in ungulates (24, 25). From that perspective, it is unlikely that all sheep from the Aggr-rewarded group were subordinate to the Photo Sheep, but that possibility cannot be excluded. Knowing the hierarchical relationships between the Photo Sheep and the tested animals, would have enabled us to clarify this point and to examine the influence of rank on learning speed.

Lastly, it is worth noting that during generalization sessions, sheep took longer to choose a branch when they made correct choices compared with incorrect choices. In juvenile pigs, similar longer response times for correct choices have been reported too (40). In this study, Nawroth et al. considered that these shorter response times were caused by impulsivity in the choice behavior of the piglets and suggested that subjects with non-impulsive approach behavior made more correct choice. They also encouraged to look at the latency to make a decision in a choice task at the individual level, rather than at the group level. Horses however have been observed to take longer to make an incorrect choice (41). This was interpreted as an uncertainty in decision making, due to awareness of the subject that it was potentially making the wrong choice. In our study, the more challenging task of transferring a rule to new cues might explain this variation, since no such difference in latency to choose was observed in the final training sessions which involved images of faces. This difference in latency to make a choice also indicates that sheep that made mistakes during the generalization sessions probably did not take time to process the two cues, but made a choice based on others factors.

### Methodological Limitations

Only 16 out of 35 sheep succeeded in reaching the second training phase, with images of faces. We allowed sheep a maximum of 180 runs in the maze to learn the task during the Colored Cards phase. This criterion is within the range of learning performances of sheep in similar tasks that also involved pairs of cues presented alternatively on both sides (80–240) (42). Given a few more training sessions, more animals might have reached the learning criterion, and so we most likely only included the faster learners in the subsequent phases of the study. Despite the preliminary phase of systematic desensitization to human handling and the habituation phase, some individuals might not have habituated fully to the experimental setup, and still considered it a stressful environment. Since stress and negative emotional states impede cognitive abilities in sheep (43, 44), it is possible that faster learners were less fearful and found repeated handling and isolation less stressful. It is also possible that these animals had better cognitive abilities. Hill breeds of sheep might also not be ideal for cognitive studies due to their high emotional reactivity; lowland breeds such as Clun Forest sheep could be more indicated for such studies It should also be mentioned that our final sample size of 16 sheep is fairly small which affects the statistical power of our study. Therefore, our conclusions may not apply to the whole population of sheep. However we did establish that sheep can discriminate between images of faces taken in situations of varying valence, and that the valence of the situation influenced learning speed.

Finally, Bovet and Vauclair (45) raised a concern about using “pictorial stimuli” in animal studies without controlling for how images are perceived by the animals. In our study, we confirmed that reactions to images of faces differed from reactions to colored cards in sheep as differences in learning speed were identified with images of faces only, and were associated with the valence of the situation in which the faces were photographed. This suggests that images of faces were perceived as faces by the sheep, but further evidence is needed to draw strong conclusions on this matter.

## Conclusion

Sheep discriminated between images of faces of conspecifics taken in an emotionally negative or neutral situation. Sheep were also able to generalize this discrimination to images of new faces, but this ability did not extend to images taken during aggressive interactions: sheep from the Aggr-rewarded group were unable to generalize the task. Learning was affected by the type of image displayed and differences in learning speed were associated with the valence of the situation in which the faces were photographed: sheep that had to associate a negative image with the reward learned faster than sheep that had to learn the neutral image-reward association. This suggests that sheep can perceive the valence of an emotional state displayed in an image of a face. This is an encouraging first step for the use of images of faces in cognitive bias studies.

## Ethics Statement

All experimental procedures were approved by the Scotland’s Rural College Edinburgh Animal Ethics Committee (Protocol no. ED-AE-2-2014). Animals were closely monitored before, during, and after the study.

## Author Contributions

LB conceived the study, collected and analyzed the data, and wrote the manuscript. HE conceived the study and contributed to the analysis of the data and writing of the manuscript. AW contributed to the conception of the study and the writing of the manuscript. AB contributed to the conception of the study and the writing of the manuscript. MH conceived the study, contributed to the data collection and analysis, and wrote the manuscript.

## Conflict of Interest Statement

The authors declare that the research was conducted in the absence of any commercial or financial relationships that could be construed as a potential conflict of interest.
